# Supplementary material for: Observation of a topological nodal surface and its surface-state arcs in an artificial acoustic crystal
Source: Nat Commun. 2019 Nov 15;10:5185. doi: 10.1038/s41467-019-13258-3 (PMC6858449; doi:10.1038/s41467-019-13258-3)
Supplement: Supplementary file 1 — Supplementary information [file 41467_2019_13258_MOESM1_ESM.pdf]

Supplementary Information

Observation of a topological nodal surface and its surface-state arcs in an artificial acoustic crystal

Yang *et al.*

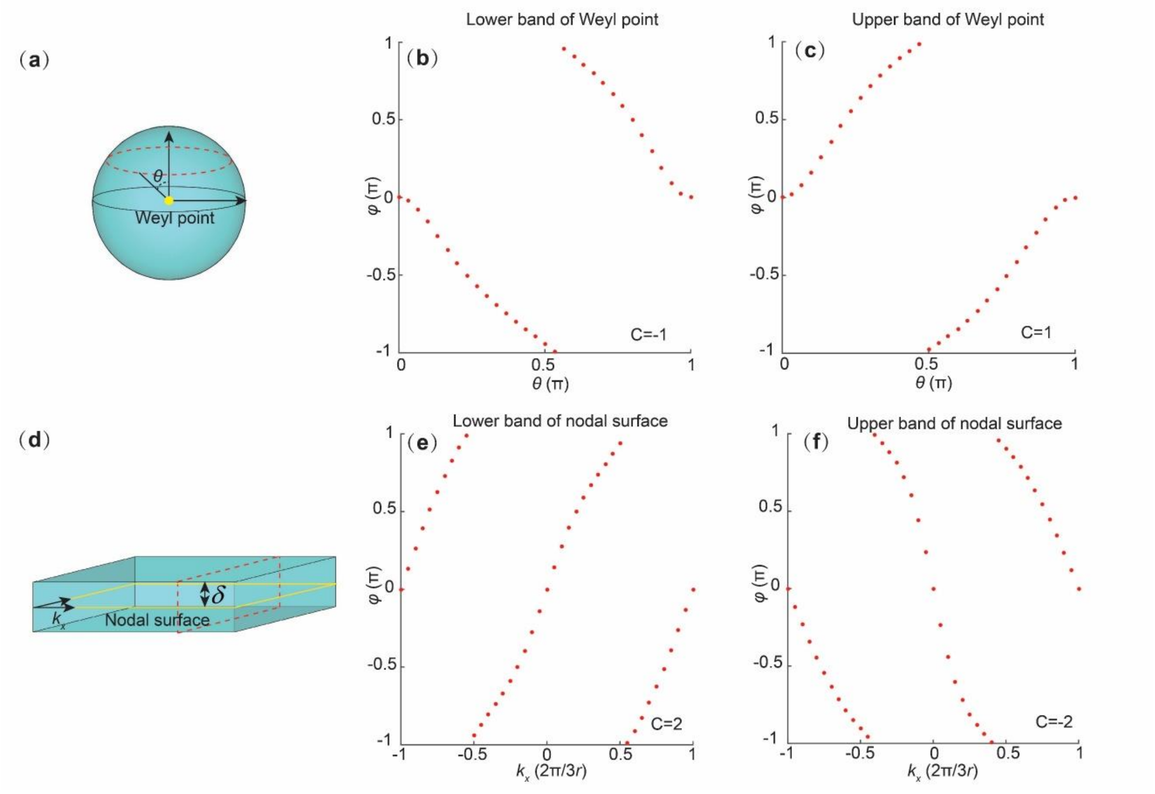

Supplementary Figure 1 | Evolution of Wannier centers on surfaces enclosing the Weyl points and nodal surface, respectively. (a) Schematic of a Wilson loop for calculating the topological charge of the Weyl point (yellow dot). (b)-(c) Wannier centers for the lower and upper bands of the Weyl point, respectively. (d) Schematic of a Wilson loop for calculating the topological charge of the nodal surface (yellow line). (e)-(f) Wannier centers for the lower and upper bands of the nodal surface, respectively.

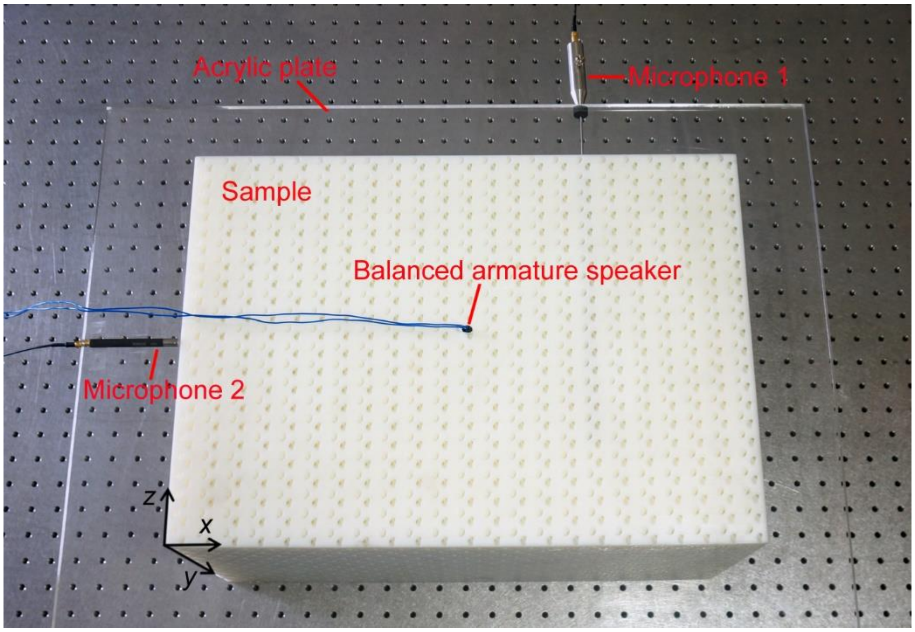

Supplementary Figure 2 | Photograph of the experimental setup for the surface state measurement. A square acrylic plate covers the measured surface of the sample. A balanced armature speaker as a source is placed at the center of the interface between the acrylic plate and the sample. Two microphones (Microphone 1 and 2) are separately placed in a sealed sleeve with a tube that penetrates deeply into the sample. During each measurement, microphone 1 is scanned point-by-point through one of the vertical air holes of the structure, to detect the input acoustic signal. Microphone 2 is fixed with respect to the sound source in order to detect the reference acoustic signal. The bulk state measurement setup is similar, and the measured plane is located in the middle of the sample.

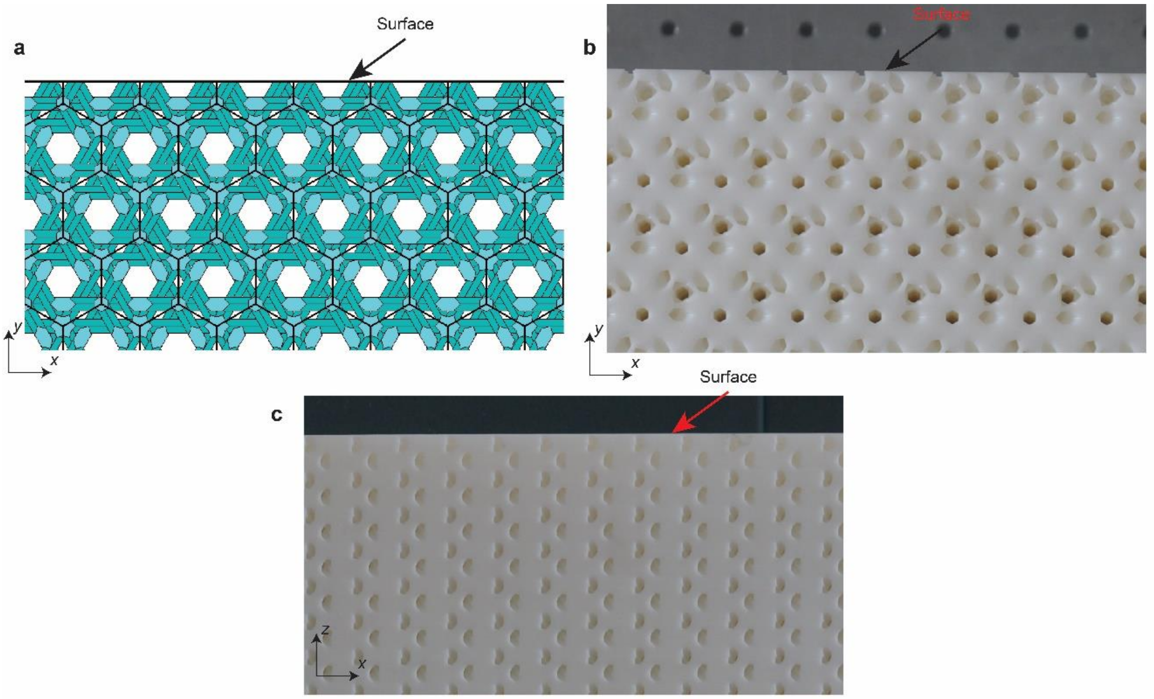

Supplementary Figure 3 | Termination surface chosen in both experiments and simulations. (a) Top view of the termination chosen. Each hexagon represents a unit cell as shown in Fig. 1(c). (b)-(c) Different views of the sample surface.

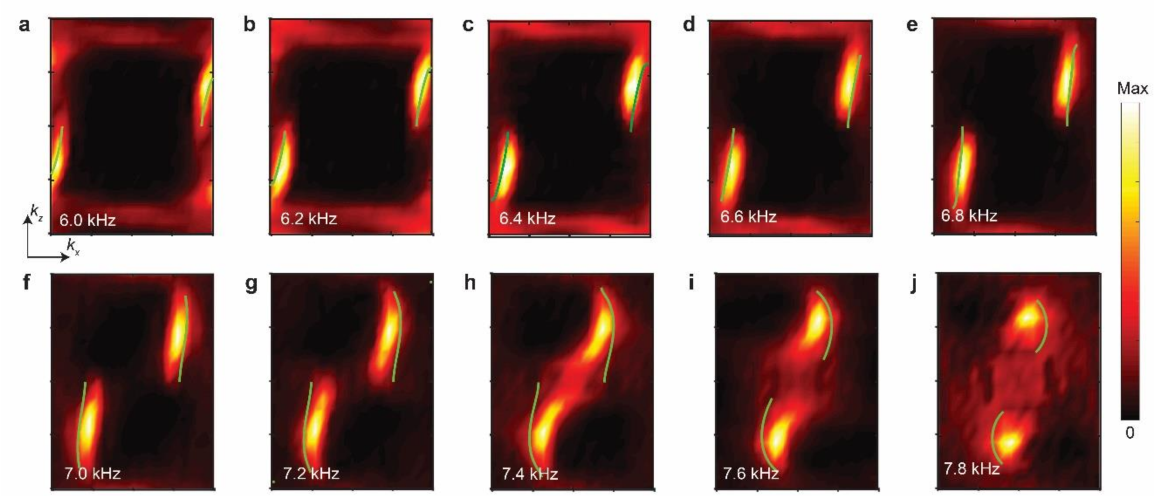

Supplementary Figure 4 | Simulated surface dispersion overlayered to the measured data. (a)-(f) Measured in-plane energy densities at frequencies ranging from 6.0 kHz to 7.8 kHz. For every plot, the horizontal axis (k_x) runs over [-π/*a*, π/*a*], and the vertical axis (k_z) runs over [-π/*h*, π/*h*]. The green lines are the simulated surface dispersion for comparison.

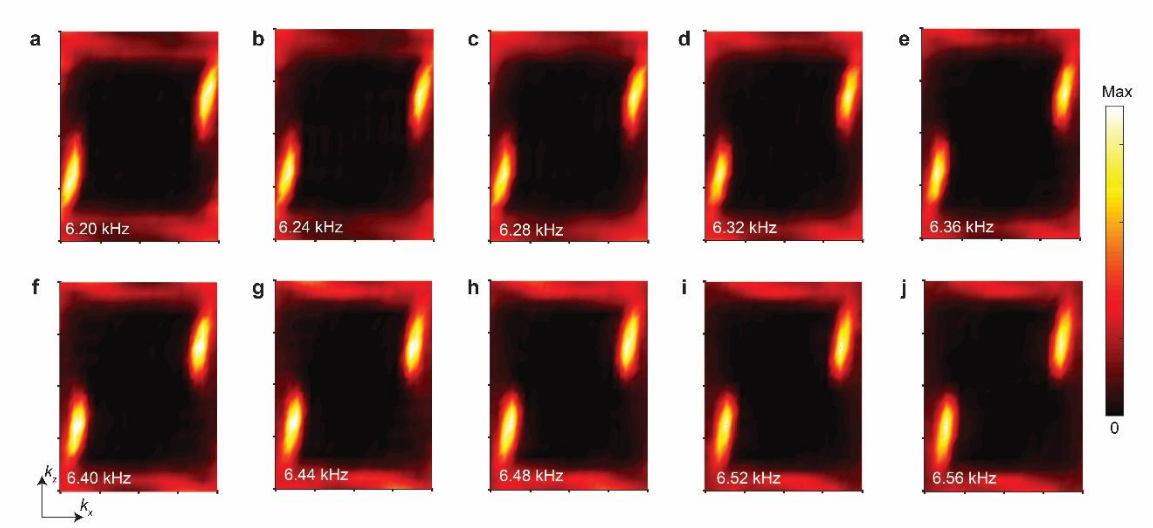

Supplementary Figure 5 | Measured in-plane energy densities at frequencies ranging from 6.20 kHz to 6.56 kHz with a frequency resolution of 40 Hz. For every plot, the horizontal axis (k_x) runs over [$-\pi/a$, π/a], and the vertical axis (k_z) runs over [$-\pi/h$, π/h].

Supplementary Note 1: Chern number calculations

The Chern numbers of the topological nodal surface and Weyl points are calculated by tracking the evolution of Wannier centers on enclosing surfaces (i.e., surfaces covering the nodal surface and the Weyl points respectively)[1]. For the Weyl points, the enclosing surface is a sphere, which is discretized into a sequence of horizontal loops, such as the red dashed line in Fig. S1(a). For the nodal surface, the enclosing surface is a hexahedron with height $2\delta=0.02\pi/h$, which we discretize into a sequence of loops of different k_x, from $-2\pi/3r$ to $2\pi/3r$. The red dashed line in Fig. S1(d) indicates one of these loops.

We calculate the Berry phase along each loop via the Wilson loop method[2], using wavefunctions extracted numerically from COMSOL Multiphysics calculations. The Wannier centers (ϕ) are the trace of the Berry phase. The calculated Wannier centers for both the Weyl point and nodal surface are shown in Fig. S1. For the Weyl point (Figs. S1(b)-(c)), the Wannier centers shift by -2π and 2π, indicating that the Chern numbers of the bands are -1 and +1, respectively. For the nodal surface (Figs. S1(e)-(f)), the Wannier centers shift by 4π and -4π, so the Chern numbers of the bands are +2 and -2, respectively.

Supplementary references

1 Chang, G. *et al.* Topological quantum properties of chiral crystals. *Nat Mater* **17**, 978-985 (2018).

2 Lu, L. *et al.* Symmetry-protected topological photonic crystal in three dimensions. *Nature Physics* **12**, 337-340 (2016).
